# Extract of white sweet potato tuber against TNF-α-induced insulin resistance by activating the PI3K/Akt pathway in C2C12 myotubes

**DOI:** 10.1186/s40529-021-00315-8

**Published:** 2021-05-18

**Authors:** Lie-Fen Shyur, Viola Varga, Chiao-Ming Chen, Shu-Chi Mu, Yu-Chih Chang, Sing-Chung Li

**Affiliations:** 1 Agricultural Biotechnology Research Center, Academia Sinica, Taipei, 11529 Taiwan; 2 School of Nutrition and Health Sciences, College of Nutrition, Taipei Medical University, 250 Wu-Hsing Street, Taipei, 11031 Taiwan; 3 Institute of Translational Medicine, Semmelweis University, 1094 Budapest, Hungary; 4 Department of Food Science, Nutrition, and Nutraceutical Biotechnology, Shih Chien University, Taipei, 10462 Taiwan; 5 School of Medicine, Fu-Jen Catholic University, New Taipei City, 24205 Taiwan; 6 Agricultural Biotechnology Research Center, Academia Sinica, Taipei, 115 Taiwan

**Keywords:** White sweet potato, C2C12 myotube, Insulin resistance, 2-NBDG, PI3K/Akt pathway

## Abstract

**Background:**

White sweet potato (WSP; *Ipomoea batatas* L. Simon No. 1) has many potential beneficial effects on metabolic control and diabetes-related insulin resistance. The improvement of insulin resistance by WSP tuber extracts on glucose uptake were not investigated in C2C12 myoblast cells.

**Results:**

WSP tuberous ethanol extract (WSP-E) was partitioned with ethyl-acetate and water to obtain ethyl-acetate layer (WSP-EA) and water layer (WSP-EW). The WSP-EA shows the highest total phenolic contents and highest antioxidant activity by Folin-Ciocalteu and (2,2-diphenyl-1-picryl-hydrazyl-hydrate, DPPH) assay, respectively. After low concentration horse serum on differentiation inducement of C2C12 myoblasts into mature myotubes, the cells were treated with TNF-α to induce insulin resistance. WSP-EA and WSP-EW extracts increased the uptake of fluorescence glucose analogue (2-[*N*-(7-nitrobenz-2-oxa-1, 3-diazol-4-yl) amino]-2-deoxy-d-glucose, 2-NBDG) in a dose-dependent manner as examined by flow cytometry. The WSP-EA enhanced glucose uptake by activation of phosphorylation of IR (pIR), IRS-1 (pIRS-1) and Akt (pAkt) involved in PI3K (phosphatidylinositol 3-kinase)/protein kinase B (Akt) pathway, also upregulated glucose transporter 4 (GLUT4) expression in myotubes.

**Conclusions:**

WSP-EA enhanced the glucose uptake in C2C12 myotubes through upregulating the PI3K/Akt pathway. The in vitro data reveal that WSP tuber extracts has potential applications to improve insulin resistance in diabetes.

## Background

Diabetes mellitus is a metabolic disorder characterized by chronic hyperglycemia resulting from defects in insulin secretion, insulin action, or both. In type 2 diabetes (T2DM), the insulin action and/or insulin secretion is impaired, latter is called insulin resistance. In adults, T2DM is the most common form of diabetes mellitus, which accounts for 90–95% of all diabetic patients (American Diabetes 2009; Maleckas et al. 2015). Therefore, the peripheral glucose uptake will be reduced, resulted in hyperglycemia. In long term high blood glucose can cause microvasular and macrovascular complications, such as atherosclerosis, nephropathy, neuropathy and retinopathy (Chawla et al. 2016).

Insulin resistance in obesity and T2DM is manifested by decreased insulin-stimulated glucose transport and metabolism in adipocytes and skeletal muscle and by impaired suppression of hepatic glucose output (Kahn and Flier 2000). Fatty acid metabolites, proinflammatory cytokines and cellular stress which destruct the insulin signaling pathway and exacerbate insulin resistance and hyperglycemia in T2DM (Boden 2011; Day and Bailey 2011). Glucose uptake via activation of the phosphatidylinositol 3‑kinase (PI3K)/protein kinase B (Akt) signaling cascade involving multiple enzymes is able to reduce glucose levels in the extracellular milieu, which in turn contributes to decrease hyperglycemia. When insulin binds to the insulin receptor (IR) on the target cell surface, causing the IR a conformational change to form phospho insulin receptor (pIR) and phosphorylation of insulin receptor substrate-1 (pIRS-1). The activation of PI3K/Akt pathway by insulin can cause GLUT4 translocated to the plasma membrane from storage vesicles and transports glucose into skeletal muscle cells (Méndez-García et al. 2018).

Oxidative stress stimulates the generation of reactive oxygen species (ROS) is believed to play an important role in developing insulin resistance in T2DM. ROS can be derived from multiple sources, such as generated during mitochondrial oxidative metabolism as well as in cellular response to inflammatory cytokines and chemokines. They contribute to induce multiple types of insulin resistance, mitochondrial dysfunction, impaired glucose tolerance, and β-cell dysfunction (Oguntibeju 2019).

Sweet potato (SP; *Ipomoea batatas* L.) belonged to the Convolvulaceae family, originated in Central Americas, is ranked the world's seventh most important crop. Extracts of sweet potato compounds have pharmacological action, clinical effect, plausible medicinal applications, and demonstrates the potential of sweet potato as a medicinal food. (Mohanraj and Sivasankar 2014). White sweet potato (WSP) extracts have antidiabetic activity in both insulin-deficient and insulin-resistant diabetic animals (Bachri et al. 2010; Kusano and Abe 2000; Oki et al. 2011). In patients with T2DM, WSP tuber extract effectively reduced insulin resistance as well as fibrinogen, fasting plasma glucose, and low-density lipoprotein-cholesterol levels (Ludvik et al. 2008, 2003). The 30% tubers of WSP had lower plasma glucose, insulin, glucose area under the curve (AUC) in diabetic mice (Shih et al. 2020), improve nutrition status and glycemic control in elderly diabetic patients (Chen et al. 2019), and reduce energy and facilitate individual weight loss (Shih et al. 2019). However, the whole WSP roots introduced to diabetic mice, resulting in an insignificant PI3K/AKT signal expression in skeletal muscle of diabetic mice.

The separation of substances that mediate or mimic the action of insulin could lead to develop of novel structures which may be of clinical use in the treatment of glucose metabolism abnormalities associated with T2DM and insulin resistance. Thus far, studies on the use of WSP tuber extracts as a functional ingredient for the management of insulin resistance cell have been scant. The aim of this study was to determine the effect of extracts of WSP on glucose uptake and explore relevant mechanism in TNF-α treated C2C12 myotubes.

## Methods

### Materials

C2C12 murine myoblast cell line was purchased from Bioresource Collection and Research Center (Hsinchu, Taiwan). Dulbecco's modified Eagle's medium, fetal bovine serum (FBS), penicillin–streptomycin-Amphotericin B (PSA), 0.5% trypsin–EDTA, and 2-[*N*-(7-nitrobenz-2-oxa-1, 3-diazol-4-yl) amino]-2-deoxy-d-glucose (2-NBDG) were obtained from Gibco-Invitrogen (Carlsbad, CA, USA). Insulin, Tumor necrosis factor-alpha, phosphatase inhibitor cocktail, 2,2-diphenyl-1-picryl-hydrazyl-hydrate (DPPH), 3-(4,5-dimethylthiazol-2-yl)-2,5-diphenyltetrazolium bromide (MTT) and other chemicals and reagents were purchased from Sigma Chemical Co. (St. Louis, MO, USA). Protein concentrations in each sample were quantified using a Bio-Rad DC Protein Assay kit (Hercules, CA, USA). The prestained protein marker for SDS-PAGE was from Bioman Sci. Co. LTD (New Taipei City, Taiwan). The antibody of β-actin, anti-phosphorylated IR, anti-phosphorylated IRS-1, anti-phosphorylated Akt, and anti-GLUT4 were purchased from cell signaling technology (Beverly, MA, USA). The band density was quantified using the analysis software Quantity One 1-D (Hercules, CA, USA).

### Preparation of sweet potato extracts and HPLC analysis of bioactive WSP-EA fraction

**Fig. 1:**
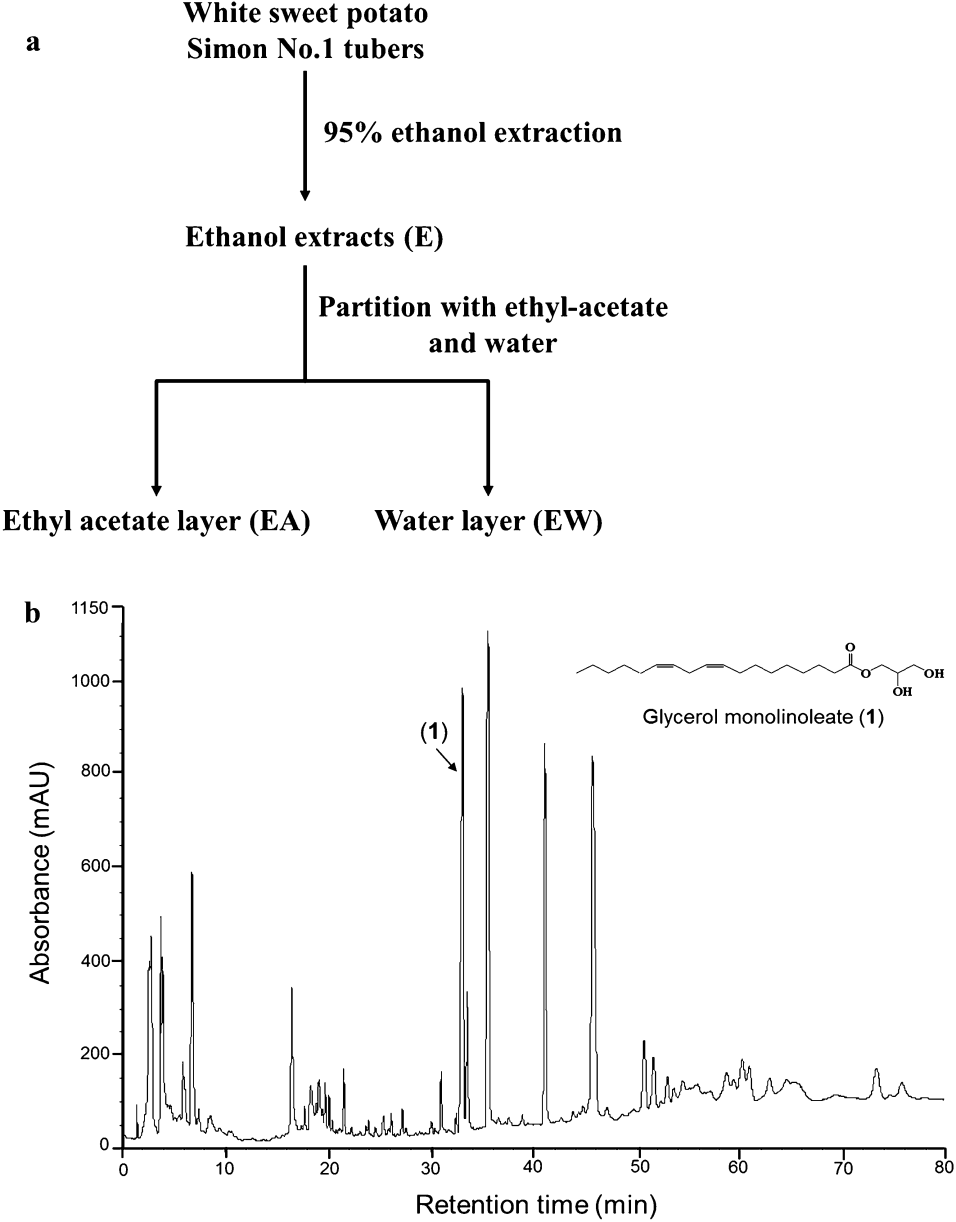
Methods for the preparation of white sweet potato tuber extracts (**A**). HPLC analysis of the most bioactive WSP-EA fraction (**B**)

Fresh mature WSP (*I. batatas* L. Simon No. 1) was harvested from a farm in the Chiayi Agricultural Experiment Station, Taiwan. About 50 kg WSP were extracted with 95% ethanol for at least 3 days to 1 week at room temperature, and repeated twice. The total WSP ethanolic extracts (WSP-E) were collected and vacuum evaporated to dry at room temperature. An amount of WSP-E was thoroughly resuspended in water and partitioned by ethyl acetate with a ratio of 1:1 to yield WSP-EA and WSP-EW fractions. The dry forms of WSP-E, WSP-EA and WSP-EW were storage at refrigerator until use. Flowchart for the preparation of WSP tuber extracts was showed in Fig. 1A. The recovery rate (%) of WSP-E, WSP-EA, and WSP-EW from fresh raw materials of Simon No.1 tubers were ~ 5.5%, 0.22% and 4.73%, respectively. We have established the chromatogram obtained by HPLC analysis of the most bioactive WSP-EA fraction and showed in Fig. 1B. The chromatogram of the sample WSP-EA was analyzed on a RP-18 column [Phenomenex Luna 5 μ C18 (2), 250 mm × 4.6 mm] using an acetonitrile (MeCN)/H_2_O gradient as the solvent system with a flow rate of 1 mL/min and measured by a UV detector at 210 nm. The HPLC gradient was MeCN (solvent B) in H_2_O (solvent A): 30% from 0 to 10 min, 70% from 10 to 20 min, 85% from 20 to 25 min, 95% from 25 to 30 min, and 100% from 30 to 80 min. Compound glycerol monolinoleate (**1**), with purity (> 97%) was purified from the WSP-EA fraction and confirmed its proton and carbon NMR spectral data with the same compound spectra published elsewhere (Okuyama et al. 2001). Compound **1** can be the index compound of the WSP-EA fraction.

### Total phenolic content

Extracts of WSP-E, WSP-EA and WSP-EW were prepared as 2 mg/mL in methanol. The use of gallic acid as a standard at a concentration from 5 to 100 mg/L according to methods of Truong et al. with some modification (Truong et al. 2007). For 0.5 mL sample plus 0.5 mL Folin-Ciocalteu reagent and mixed with 50 µL of 10% sodium-bicarbonate. After 1-h incubation at room temperature, OD values were measured at 735 nm in plate reader. Results were expressed as milligrams of gallic acid equivalent (GAE) per gram by dry weight (mg GAE/g DW).

### In vitro DPPH radical-scavenging activity

DPPH is a radical-generating substance widely used to monitor the free radical-scavenging abilities of various antioxidants. The evaluation of free radical scavenging ability was according to Yamaguchi et al. (1998). Various WSP extracts were prepared and tested their antioxidant activity at a range of concentrations from 5 to 250 μg/mL. Ascorbic acid was used as a standard at a concentration ranged from 0.078–10 mg/mL. 400 μL Tris–HCl buffer (pH 7.4, 100 mM) and 500 μL of DPPH (500 μM) solution were added, samples were shaken vigorously and left to stand for 20 min at room temperature in the dark. OD values were measured at 517 nm using plate reader. The radical scavenging activity was measured as a decrease in the absorbance of DPPH and was calculated using the following equation: scavenging effect (%) = (1 − A_Sample (517 nm)_/A_Control (517 nm)_) × 100.

### Differentiation of C2C12 cells and MTT assay

C2C12 myoblasts were maintained in a humidified atmosphere containing 5% CO_2_ at 37 °C using DMEM-high glucose with 20% FBS supplemented with PSA antibiotics to form postconfluence within 2–3 days. For inducing differentiation, DMEM with high glucose plus 2% horse serum was added. Medium was changed every 1 day up to the three days’ time point and after that, every 12 h medium supplement. As these cells differentiate, they begin to deplete and acidify the medium more quickly. To determine the cytotoxic dosage of the WSP extracts used in subsequent experiments, C2C12 myoblasts were plated using 96-well plate (200 μL medium per well) and cell densities of 10,000 cells/mL. After differentiation, WSP extracts (ranged 12.5–200 µg/mL) will be added to the new medium. Cells will be incubated for 24 h at 37 °C, 20 μL of MTT Solution (5 mg/mL) was added per well and incubate for 3 h at 37 °C, then 100 μL of the DMSO was added and product can be quantified by spectrophotometry using a plate reader at 570 nm.

### Evaluation of insulin resistance by glucose uptake assay

2-NBDG is a fluorescence glucose analogue used to estimate the amount of glucose uptake by flow cytometry and thereby to explore the regulation of glucose metabolism and mechanism of insulin resistance (Lee et al. 2011). Briefly, myoblasts were differentiated and insulin resistance was induced by TNF-α (20 ng/mL) induction for 24 h. Then the myotubes were incubated with Krebs–Ringer bicarbonate (KRB) buffer containing 160 µM 2-NBDG and 500 nM insulin with or without SP extract (12.5–200 µg/mL) and 50 µM thiazolidinediones (TZD) as a control for insulin-sensitizing drugs (Reginato and Lazar 1999) and incubation at 37 °C for 30 min in the dark. The TZD is a diabetic drug that can improves insulin signaling in human adipocytes, as evidenced by increasing insulin-induced PI3 kinase and Akt activities (Lin et al. 2005). The reaction was stopped by washing cells with ice-cold phosphate buffered saline (PBS), then transferred cells into glass tubes. Samples were analyzed using a FACSCalibur flow cytometer (San Jose, CA, USA) by 20,000 cells per sample. The excitation and emission wavelengths of 488 and 542 nm, and analyzed using Cell Quest Pro software. The intensity of fluorescence reflects the uptake of 2-NBDG in the cells.

### Western blotting

The TNF-α induced C2C12 myotubes were treated with different WSP extracts and TZD were homogenized in a modified RIPA buffer (0.5 M Tris–HCl at pH 7.4, 1.5 M sodium chloride, 2.5% deoxycholic acid, 10% NP‐40, and 10 mM EDTA) and 10% protease and phosphatase inhibitor cocktail. The homogenates were centrifuged at 10,000*g* at 4 °C for 15 min, and the supernatants were taken as the cell extract. For demonstrating GLUT4 expression in the membrane, the cell lysate was prepared using a Mem-PER kit from Thermo Fisher Scientific (Waltham, MA, USA) to enrich the membrane and cytosolic proteins. Protein concentrations in each sample were quantified using a commercial assay kit with bovine serum albumin as a standard. Equal amounts of proteins (40 μg) were mixed with 4X SDS-PAGE loading buffer, containing 200 mM Tris–Cl (pH 6.8), 400 mM DTT, 8% SDS, 0.4% bromophenol blue and 40% glycerol, and boiled for 10 min in water. The denature samples were subjected to 10% sodium dodecyl sulfate–polyacrylamide gel electrophoresis and then transferred onto a polyvinylidene difluoride transfer membrane. The blots were blocked with Tris-buffered saline with 0.1% Tween^®^ 20 Detergent (TBST) containing 1% BSA for 1 h, then washed with TBST three times, and incubated with 1:2000 diluted solutions of anti-pIR, anti-IR anti-pIRS-1, anti-pAkt, anti-Akt, and anti-GLUT4 antibodies overnight at 4 °C. The β-actin antibodies as staining used as a control to ensure equal protein loading in each lane of the gel. The membrane was washed three times each for 5 min in TBST, and then shaken in a solution of anti-mouse IgG or anti-rabbit IgG secondary antibodies. After repeating the washing step, the binding of antibodies was determined using FAST 5-bromo-4-chloro-3-indolyl phosphate/nitro blue tetrazolium as the substrate of the secondary antibody-conjugated alkaline phosphatase. The band density was quantified using the analysis software Quantity One 1-D by Bio-Rad Laboratories, Inc. (Hercules, CA, USA).

### Statistical analysis

Every experiment was performed in triplicate and the mean and data are represented as mean ± standard deviation (SD). Statistical evaluation was performed using one-way ANOVA, followed by Tukey’s post-hoc test. All data analyses were performed using SPSS (version 19; SPSS Inc., Chicago, IL, USA). Differences were considered significant at P < 0.05.

## Results

### Total phenolics and antioxidant activity

**Fig. 2:**
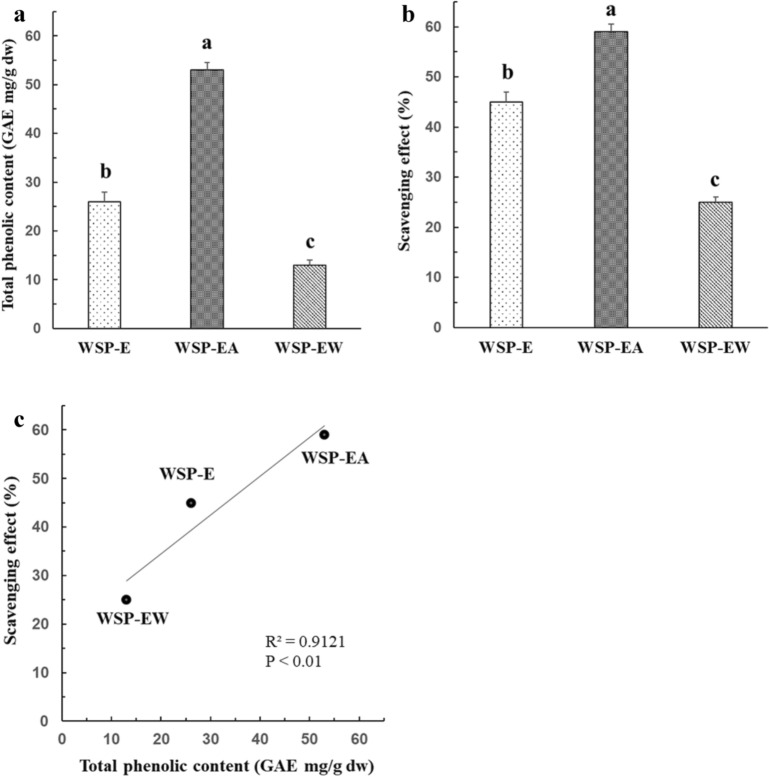
Total phenolic content, DPPH radical-scavenging activity and relationship between antioxidant activity and total phenolic content in WSP. **A** Total phenolics of sweet potato extracts. **B** Percentage scavenging activity of sweet potato extracts. **C** Correlation between % scavenging activity and total phenolic contents. *GAE* gallic acid equivalent, *WSP-E* white sweet potato ethanol extract, *WSP-EA* white sweet potato ethyl-acetate fraction, *WSP-EW* white sweet potato water fraction. Values were presented as mean ± SD of 3 independent experiments performed in triplicate, and were analyzed using one-way ANOVA, followed by a post hoc Tukey’s test for multiple comparisons. Different letters above the error bars indicate significant differences among treatments (*P* < 0.05)

The total phenolic content, measured by Folin-Ciocalteu assay is shown in Fig. 2A. WSP-EA extract is the most abundant in total phenolics, with a value of 52.6 ± 2.9 mg/g of GAE, followed by WSP-E (26.1 ± 1.8 mg/g of GAE), and WSP-EW (12.6 ± 0.8 mg/g of GAE), respectively. The polyphenol content of WSP-EA was about 4 times that of WSP-EW. The free radical scavenging ability of the extracts by DPPH assay was presented in Fig. 2B. The data showed that, at 50 μg/mL, WSP-EA has the highest scavenging activity with 58.0 ± 4.3% followed by WSP-E with a value of 44.5 ± 3.8 then the lowest was WSP-EW (24.9 ± 1.4%). The total phenolic contents and free radical scavenging activity revealed a significant positive correlation (R^2^ = 0.9121, p < 0.001) was shown in Fig. 2C. We have carried out additional experiment at a concentration range from 5 to 250 μg/mL of the WSP extracts to determine their anti-oxidant activities. The IC_50_ values for scavenging DPPH radicals by WSP-E and WSP-EA were determined at 175 and 39 μg/mL, respectively. WSP-EW only inhibited ~ 30% of DPPH radicals at 250 μg/mL.

### Cell viability assay

**Fig. 3:**
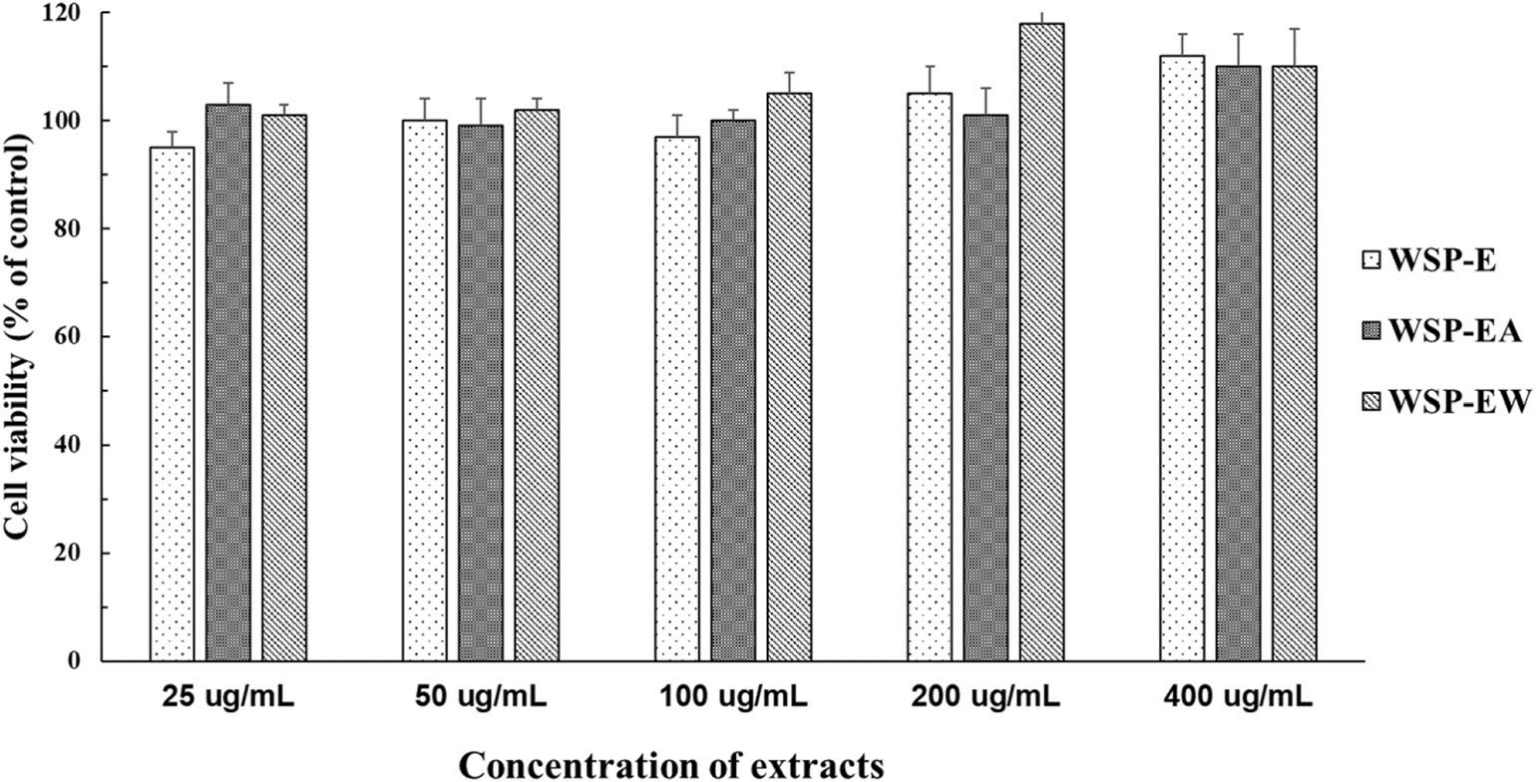
C2C12 myotubes viability evaluated by MTT assay in different WSP extracts and concentration from 25 µg/mL to 400 µg/mL for 24 h at 37 °C. *WSP-E* white sweet potato ethanol extract, *WSP-EA* white sweet potato ethyl-acetate fraction, *WSP-EW* white sweet potato water fraction. Values were presented as mean ± SD of 3 independent experiments performed in triplicate, and were analyzed using one-way ANOVA, followed by a post hoc Tukey’s test for multiple comparisons

The cell viability of C2C12 myoblasts by different WSP extract treatments were accessed by MTT assay. Cells were treated with different concentration ranged between 25 and 400 µg/mL for 24 h. The result is shown on Fig. 3. There was no significantly difference between each group. In other words, these extracts did not cause any toxicity any toxicity up to 400 µg/mL concentration on C2C12 muscle cells.

### Glucose uptake assay using flow cytometry

**Fig. 4:**
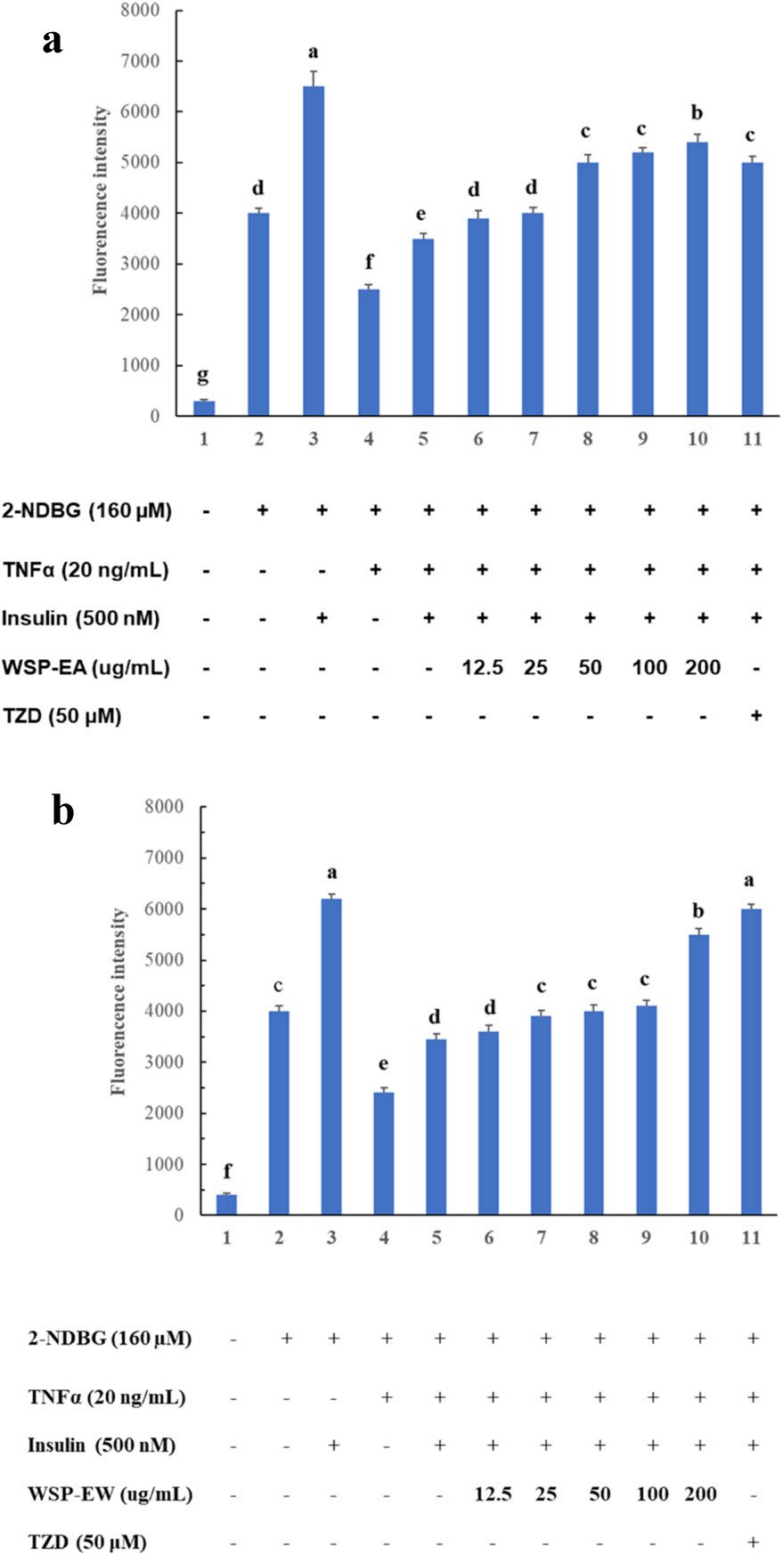
Glucose uptake evaluation of different concentration of WSP extracts on C2C12 myotubes. C2C12 cells were differentiated to myotubes in DMEM-high glucose plus 2% horse serum, further insulin resistance was induced by TNF-α (20 ng/mL) for 24 h. The different concentration of WSP-EA fraction (12.5 µg/mL to 200 µg/mL) (**A**) and WSP-EW fraction (**B**) were used to quntified the uptake of glucose analog 2-NBDG by flow cytometry. *TNF-α* tumor necrosis factor alpha, *WSP-EA* white sweet potato ethyl-acetate fraction, *WSP-EW* white sweet potato water fraction. *TZD* thiazolidinedione. Values were presented as mean ± SD of 3 independent experiments performed in triplicate, and were analyzed using one-way ANOVA, followed by a post hoc Tukey’s test for multiple comparisons. Different letters above the error bars indicate significant differences among treatments (*P* < 0.05)

TNF-α could cause muscle cell inflammation and lead to insulin resistance. Our data revealed treatment TNF-α 20 ng/mL for 24 h could significantly reduce 2-NBDG uptake by C2C12 myotubes. Differentiated C2C12 cells significantly increased glucose uptake under the action of insulin. However, cells treated with TNF-α for 24 h significantly reduced fluorescence glucose uptake by nearly 44–46% under the action of insulin (Fig. 4A, B). It showed that myotubes have successfully induced insulin resistance. Treat myotubes with different concentrations of WSP-EA (12.5–200 µg/mL), we found the fluorescence intensity increased significantly with the increase in concentration by a dose dependent manner (Fig. 4A). In addition, the differentiated cells treated with WSP-EW also increased their fluorescence intensity only in a higher dosage, but not showed a dose dependent manner (Fig. 4B). Further evaluation of insulin resistance in myotubes, both highest concentration of WSP-EA and WSP-EW significantly increase the glucose uptake by 54% and 59%, individually.

### Western blotting findings of WSP-EA

**Fig. 5:**
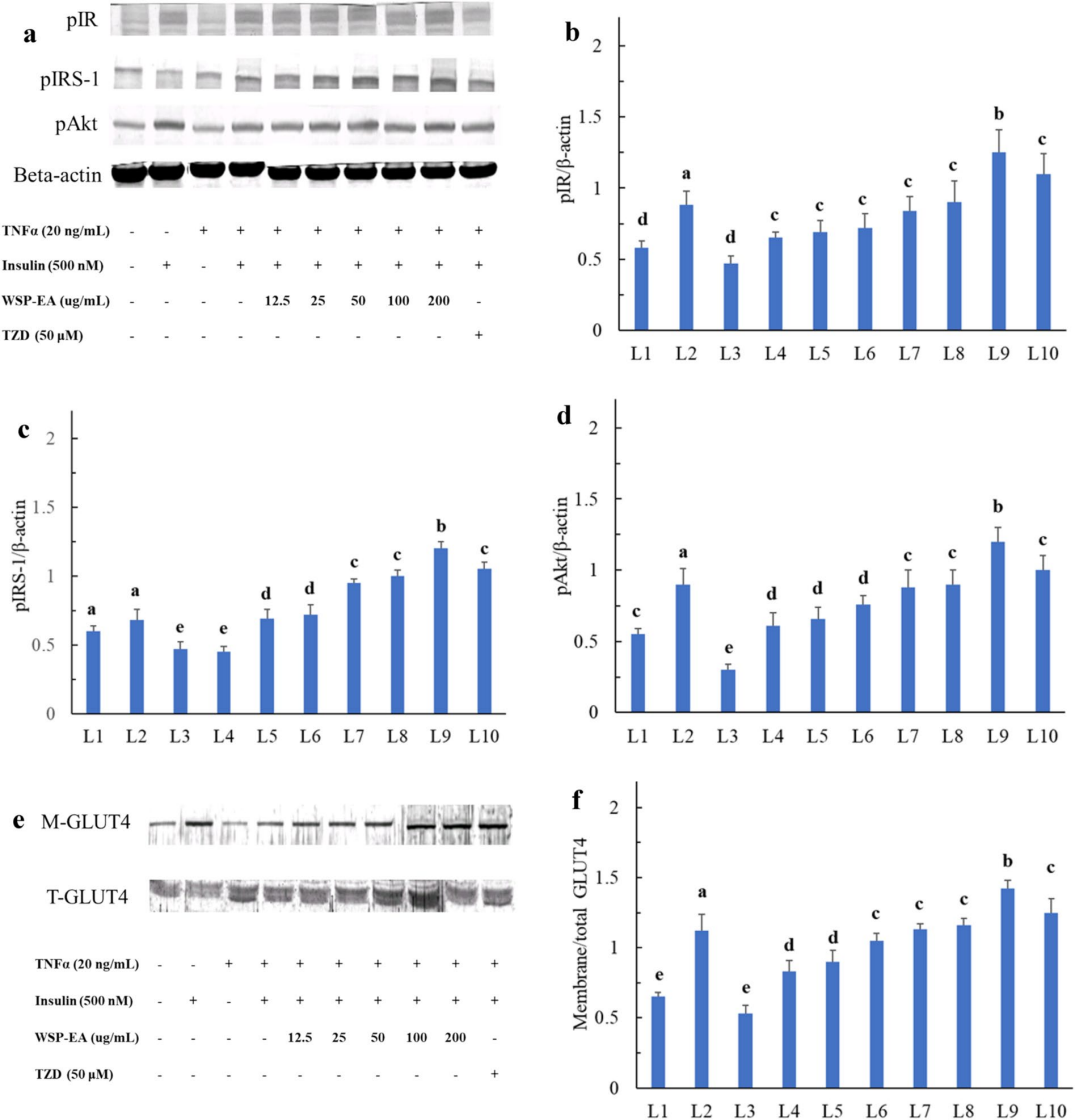
Effect of WSP-EA extracts on insulin-stimulated phosphorylation of the PI3K/Akt pathway. **A**–**F** Western blotting of insulin-stimulated phosphorylation of pIR, pIRS-1, pAkt and M-GLUT4 expression. *TNF-α* tumor necrosis factor alpha, *ins* insulin, *WSP-EA* white sweet potato ethyl-acetate fraction. *TZD* thiazolidinedione, *pIR* phosphorylation of insulin receptor, *pIRS-1* phosphorylation of insulin receptor substrate 1, *pAkt* phosphorylation of protein kinase B, describe the scale of y-axis of each quantification: membrance glucose tranporter 4, *T-GLUT4* total glucose transporter 4. Values were presented as mean ± SD as independent experiments performed in triplicate, and were analyzed using one-way ANOVA, followed by a post hoc Tukey’s test for multiple comparisons. The scale of y-axis of each quantification means ratio of candidate phosphorylated-protein expression to house keeping protein (**B**–**D**) or ratio of M-GLUT4 to total GLUT4. Different letters above the error bars indicate significant differences among treatments (*P* < 0.05)

We analyzed the protein expression to determine whether WSP-EA extracts increased glucose uptake by activating the PI3K/Akt pathway. Our data revealed that protein expression of phosphorylation of IR (pIR), IRS-1 (pIRS-1) and Akt (pAkt) significantly decreased after treated with TNF-α for 24 h. When C2C12 myotubes were treated with different concentrations of WSP-EA (12.5–200 µg/mL), the expression of pIR, pIRS-1 and pAkt increased significantly in a dose-dependent manner (Fig. 5B–D). We did not see effect on the protein levels of IR, IRS-1 and Akt in the cells after treating WSP-EA extracts by western blotting (data not shown), we therefore only show the result of phosphorylated IR, IRS-1 and AKT proteins in Fig. 5. In addition, to separate of cytosolic and membrane proteins from whole cells and measure the GLUT4 expression in different concentrations of WSP-EA (12.5–200 µg/mL), we also found that GLUT4 translocation to membrane was significantly upregulated with increasing concentration. It demonstrated that increased membrane GLUT4 expression by insulin action and decreased by TNF-α treatment. WSP-EA extract could successfully recover this decrease even in low concentration (Fig. 5E).

## Discussion

WSP have claimed to have antidiabetic properties according to the traditional medicine in Taiwan. They contain a lot of fibers, minerals, β-carotene and polyphenols. Polyphenols had shown in many studies to exert positive effects for the prevention of metabolic disorders such as diabetes. Thus, the antidiabetic effect of WSP-EA may be related to their phenolic composition, which comprises several major compounds, including flavonols, anthocyanins, caffeic acids, chlorogenic acids, quercetins, myricetins, apigenins and luteolins (Ishiguro et al. 2007; Truong et al. 2007). As polyphenols in general are moderately water-soluble, the antidiabetic effect of the water fractions can be due to other compounds. Ayeleso et al. demonstrated extract of orange sweet potato showed the presence of polyphenols which ameliorate oxidative stress and modulate T2DM associated genes in insulin resistant C2C12 cells (Ayeleso et al. 2018). In the present study, we found WSP-E, WSP-EA and WSP-EW have high total polyphenol content and scavenging effect of DPPH. The result confirmed that WSP extracts exerts its anti-diabetic properties in TNF-α treated C2C12 myotubes by glucose uptake assay, both high dosage of WSP-EA and WSP-EW increased the uptake of 2-NDBG than TNF-α along significantly.

The total phenolic content of sweet potatoes depends on the geographic area, weather, storage conditions, preparation methods and genotype (Ishiguro et al. 2007). Latest is the most widely described, and there are some articles comparing different genotypes with different root color. For example Makori et al. also compared flesh of white (Simon No. 1 and Shangshu 19), purple (Yuzi No. 7) and orange (Pushu 32) sweet potatoes, and found that purple sweet potatoes (9.8 mg/g of GAE) contained approximately 2 times more polyphenols thane orange (5.7 mg/g of GAE) and white (5 mg/g of GAE) phenotypes on a dry weight basis (Makori et al. 2020). Compare to our results, the WSP-EA extract had very high level of total phenolics with 52.6 ± 2.9 mg/g of GAE. The scavenging activity of the samples showed a strong correlation with the total phenolic content, which is consistent with previous studies (Truong et al. 2007). In the present studies, WSP-EA had the highest scavenging activity with 58.0 ± 4.3%, showed that the scavenging activity is mainly due to aboudant polyphenols in WSP-EA. In addition, the WSP-E crude extract also had higher scavenging activity than WSP-EW due to the solubility of polyphenol. The reactive oxygen species can disrupt intracellular signalling pathways, thereby dysregulating the expression of genes associated with insulin secretion and signalling. However, the various polyphenols and phenolic compounds exhibit remedial benefits involved in the T2DM by modulate insulin resistant process have not yet been properly elucidated (Kang et al. 2019). Therefore, our result points out that WSP-EA in a dose dependent manner exerted fluorence glucose uptake. The protein expression levels of pIR, pIRS1 and pAkt upregulated with the increase of WSP-ER intervention, suggesting that phenolic compounds of WSP-EA has an antidiabetic effect via activation of the PI3K/Akt pathway and increase GLU4 translocation to the plasma membrane.

There are numerous animal studies evaluating the side effects of different sweet potato treatments, but there are only a few papers evaluating their toxicity in cell culture. These papers are mostly cancer studies, where cytotoxicity by apoptotic pathway is beneficial. For example the extract from baked sweet potato showed cytotoxicity against human myelocytic leukemia HL-60 cells (Rabah et al. 2004). The anthocyanins from purple sweet potatoes have the strongest antioxidant ability among polyphenols, therefore they are widely studied. In in vitro they showed to exert protective effect in chemically induced toxicity according to the study of Hwang et al. (2011). In other study purple sweet potato fermented milk prevented the cell death of macrophage-like RAW264.7 cells (Wu et al. 2012).There was no literature regarding cytotoxicity of white and orange sweet potatoes on healthy cells, such as C2C12 cells. As a very important crop root it is consumed in high amount in some regions (Champagne et al. 2009), so it is not surprising that the safety of this food and for the extracts is also very high. According to our unpublished data in FL83B hepatocytes, 400 µg/mL concentration was also the upper limit for the WSP extracts without significant toxicity.

Measuring the uptake of 2-NBDG glucose analogue is a very sensitive and trustable method for the first-line evaluation of the antidiabetic drugs and agents. This is a good method for measuring glucose uptake by different treatments; and already described in some studies using skeletal muscle cells. For example the effect of tangeretin (Kim et al. 2012) and citrus junos Tanaka peel extract (Kim et al. 2013) and Monascus sp. (Lee et al. 2011) were also tested in the same model system and found increased glucose uptake in C2C12 myotubes. However, the first two papers were focusing on AMPK pathway, while monascus showed the effect on the PI3K pathway by using 2-NBDG uptake with insulin-dependent glucose uptake in muscle cells. Following its methodology, we also found increased glucose uptake by WSP treatments. In addition, the L6 rat skeletal muscle cells were used to test the effect of guanidinium derivatives (Yang et al. 2013) and p-Coumaric acid (Yoon et al. 2013) on glucose uptake, and they both found the involvement of the AMPK pathway. AMPK can be activated by exercise to induce glucose uptake to cover the elevated energy demand of the muscles. In C2C12 cells electrically stimulated contraction can increase 2-NBDG uptake, which is independent of insulin and can mimic the effect of exercise (Kaji et al. 2010). Other authors also introduced 2-NBDG to analyze the contraction-mediated signals, calcium and AMPK, on glucose uptake under acute and chronic conditions (Park et al. 2009).

GLUT4 is the insulin-regulated glucose transporter found primarily in adipose tissues and skeletal muscle. The glucose uptake by GLUT4 involves the translocation of the GLUT4 containing vesicles to the plasma membrane. This trafficking was shown by separating the membrane proteins from the cytosolic proteins, which showed elevated GLUT4 levels by the WSP-EA extracts. It had been proposed that the phosphorylation of AS160 (Akt substrate, 160 kDa), a Rab GTPase-activating protein play an important role in the regulation of GLUT4 translocation (Randhawa et al. 2008). AS160 seems to be the common effector of AMPK and Akt, which is a downstream effector of PI3K. To investigate, which pathway play major role in WSP-EA induced glucose uptake, we also performed western blot using anti-Akt and anti-AMPK antibodies. Unfortunally, AMPK have not differed significantly in any of the groups (data not shown), while Akt showed dose dependent activation by WSP-EA treatments. So, we speculated that the glucose uptake is activating the PI3K/Akt pathway, but not the AMPK pathway. Akt is involved in multiple cellular processes such as glucose metabolism, apoptosis, cell proliferation, transcription and cell migration. As a member of the insulin signaling pathway, it is required to induce glucose transport. This protein kinase is activated by insulin involving PI3 kinase activation. Akt can be activated by at Thr308 by PDK1 and by phosphorylation within the carboxy terminus at Ser473. Phosphorylation on both sites is required for full activation of Akt (Hill et al. 2001).

To further dissect the role of PI3K pathway in the effect of sweet potato, we analyzed the expression levels of p-IR and p-IRS-1. TNF-α plays a central role in the state of insulin resistance associated with obesity. Paz et al. had showed that one important mechanism by which TNF-α interferes with insulin signaling is through the serine phosphorylation of IRS-1, which can then function as an inhibitor of the tyrosine kinase activity of the IR (Paz et al. 1997). Therefore, agents which can increase insulin-induced Tyr phosphorylation, or block Ser/Thr—phosphorylation caused by TNF-α, can increase the ability of IRS-1 to interact with the juxtramembrane region of IR. We could observe the elevation of the Tyr phosphorylation in the effect of WSP-EA and TZDs, giving additional evidence for the involvement of the PI3K pathway.

In T2DM, overweight patients are not only high serum free fatty acids and hyperinsulinemia, but also increased leptin, MCP-1, IL-6, and TNF-α production by adipocytes. Oxidative stress cause by NADPH oxidase and possibly adipocyte mitochondria can alternate intracellular signaling that leads to the formation of insulin resistance (Maslov et al. 2018). Plant phytochemicals, like polyphenols, β-carotene and anthocyanins are extensively studied for their ability to scavenge the free radicals and therefore attenuate the effect of ROS-inducing agents. Therefore, we cannot exclude several possible mechanisms, including the inflammatory process involving on nuclear factor-kappa B (NF-κB) and c-jun terminal NH2-kinase (JNK) signaling pathways. For example, astaxanthin increased IRS-1 tyrosine and Akt phosphorylation and a decrease JNK and IRS-1 serine 307 phosphorylation in L6 cells (Ishiki et al. 2013), or resveratrol upregulated Nrf2 expression to attenuate methylglyoxal-induced insulin resistance in Hep G2 cells through the extracellular signal-regulated kinase (ERK) pathway but not the p38 or c-Jun N-terminal kinase (JNK) pathways (Cheng et al. 2012). β-carotene, the major pigment of orange sweet potatoes, could reverse the ROS inducing effect of TNF-α in 3T3-L1 adipocytes during differentiation by enhancing gene expressions of adiponectin, adipocyte lipid-binding protein, GLUT4 and peroxisome proliferator-activated receptor-gamma2 (Kameji et al. 2010). In addition, many phenolic compounds have been described in sweet potatoes, but their exact mechanism and interactions with other compounds are different influence in diabetic conditions. For this reason, there are further studies should be done to evaluate the anti-inflammatory and antidiabetic actions of each sweet potato compounds.

## Conclusion

Sweet potatoes contain a lot of active compounds, especially high in polyphenols, which can have good antioxidative properties and potential applications to improve insulin resistance in diabetes. WSP extracts helped the glucose uptake in TNF-α treated C2C12 cells. The improvement of WSP-EA against TNF-α-induced insulin resistance was mainly mediated by PI3K/Akt pathway. This was demonstrated by the IR, IRS-1 and Akt activation by phosphorylation, that might further upregulate GLUT4 expression. Although the PI3K inhibitor assay were not employed in this study, the WSP-EA intervention showed a dose-dependent upregulation in good agreement with the positive control drug TZD. This could explain that the up-regulation of GLUT4 expression in C2C12 myotubes is activated by insulin signaling pathways and could be regulated by WSP-EA. Overall, we can conclude that sweet potato extract can exert beneficial effect in diabetic conditions in vitro. The pharmacokinetics and bioavailability studies in animals that are essential in the design of safe dosage regimens of the extracts and/or its active ingredients.

## Data Availability

All data generated during the study are interpreted in the manuscript.
